# Characteristics of the patellofemoral joint of patients with DDH and the effects of Bernese periacetabular osteotomy on the patellofemoral joint

**DOI:** 10.1186/s12891-022-05291-z

**Published:** 2022-04-08

**Authors:** Jianping Peng, Fei Xiao, Junfeng Zhu, Chao Shen, Yang Li, Xiuguo Han, Yimin Cui, Xiaodong Chen

**Affiliations:** Department of Orthopaedics, Xinhua Hospital Affiliated to Shanghai Jiao Tong University School of Medicine, Building 8, No.1665, Kongjiang Road, Shanghai, 200092 China

**Keywords:** Bernese periacetabular osteotomy, Developmental dysplasia of the hip, Anterior knee pain, Patellofemoral joint

## Abstract

**Background:**

Some patients with developmental dysplasia of the hip (DDH) complained of anterior knee pain (AKP) before and after Bernese periacetabular osteotomy (PAO) surgery. The purpose of this study was to (1) identify the characteristics of patellofemoral joint (PFJ) deformities in patients with DDH and (2) to determine the effects of PAO on the PFJ.

**Methods:**

Seventy patients (86 hips) were included in the DDH group. Thirty-three patients (33 knees) without AKP and hip pain were included in the control group. All patients underwent simultaneous CT scans of the hip and knee joints before PAO and after hardware removal surgery. The distance from the anterior inferior iliac spine to the ilioischial line (DAI), was measured in DDH patients. Imaging parameters of knees, including the sulcus angle (SA), femoral trochlear depth (FTD), patellar width (PW), tibial tuberosity-trochlear groove (TT-TG), patellar tilt angle (PTA) and lateral shift of the patella (LSP) were measured in patients in both the DDH and control group. TT-TG, PTA, and LSP of DDH patients were measured before PAO and after hardware removal. The DAI, PTA, LSP and TT-TG of all DDH patients before and after Bernese PAO were compared using paired t-tests. The FTD, PW, and SA of the DDH patients and the control group were analyzed using independent t-tests. PTA, TT-TG, and LSP between the control group and preoperative DDH patients, between the control group and post PAO patients were compared using independent t-tests.

**Results:**

The DAI changed from 4.04 ± 0.61 mm before PAO surgery to 5.44 ± 0.63 mm after PAO surgery. The SA of the DDH group (140.69 ± 11.30 degree) was greater than that of the control group (130.82 ± 6.43 degree). The FTD and the PW of the DDH group (5.45 ± 1.59 mm, 4.16 ± 0.36 mm) were smaller than that of the control group (7.39 ± 1.20 mm, 4.24 ± 0.38 mm). The changes in LSP, PTA, and TT-TG before and after surgery were not statistically significant. Both before and after PAO, there was no statistically significant difference in the parameters of LSP, PTA, and TT-TG compared with the control group.

**Conclusion:**

The knee joints of DDH patients presented a certain degree of femur trochlear groove dysplasia and patellofemoral instability. PAO surgery did not change PFJ stability, although the origination point of the rectus femoris muscle moved laterally during PAO surgery.

## Background

Developmental dysplasia of the hip (DDH) is one of the most common causes of secondary osteoarthritis (OA) because of insufficient coverage of the femoral head and subsequent abnormal overload of the acetabular rim [1]. When left untreated, this structural and mechanical abnormality can lead to progressive hip degeneration and eventual end-stage OA. Bernese periacetabular osteotomy (PAO), introduced by Ganz [2] and his colleague in 1988, has become one of the most popular surgical procedures to treat DDH. This procedure can improve acetabular coverage, optimize the hip rotation center, reduce stress on the articular cartilage, and prevent or postpone hip OA progression. This hip preservation procedure has several advantages: requiring only a single incision; maintaining posterior column integrity to provide inherent stability; preserving the shape and size of the true pelvis to allow normal child delivery in female patients; and maintaining the acetabular blood supply, allowing for better correction of the acetabular position [2–4].

Since Bernese PAO was originally introduced, numerous studies have reported excellent radiographic outcomes, marked pain reduction, and functional improvement in patients [2–7]. So far, the longest follow-up time for the periacetabular osteotomy in Bern has been 30 years [4]. The conversion rate to total hip arthroplasty (THA) or arthrodesis was 12.4% at 10 years for the first series reported by Ganz [2]; notably, 60% of hips were preserved at 20 years post-PAO [3], and 29% of hips were still free from conversion to THA at 30 years post-PAO surgery [4]. Another long-term result from Millis’s center showed a cumulative survival of 76% at the 10-year follow-up and 74% at the mean 18-year follow-up; moreover, 53% remained asymptomatic and did not meet any failure criteria at the most recent follow-up [5]. The prospective minimum 2-year patient-reported outcomes from the multicenter academic network for conservational hip preservation outcomes research (ANCHOR) group showed a 99.2% hip survival rate and a 93% early satisfaction rate [6]. In our retrospective cohort, 40 hips (in 41 patients) survived at the average 5.1-year follow-up, and the Harris hip score (HHS) improved from 63.7 to 88.4 by this follow-up point [7]. The latest review, including 4070 hips of 40 studies with a mean follow-up period of 52.8 months, demonstrated good to excellent outcomes in 82% [8]. The majority of patients had dramatic pain relief [9] and substantially improved daily function [10, 11]. Even patients with mild arthritis or patients older than 40 years [12] could achieve a certain levels of pain relief and functional improvement.

Some complications are inevitably associated with this procedure. Major complications were noted in 6 to 37% of patients after PAO surgery [12]. Ali et al. recently identified an overall complication rate of 18.3%: 11.2% were Clavien grades I and II, and 7.1% were grades III and IV [8]. The ANCHOR group documented a 5.9% risk of Clavien grade III and IV complications after the learning curve for surgeons experienced with PAO [13]. The most common complications include lateral femoral cutaneous nerve (LFCN) neurapraxia, wound-related issues, heterotopic ossification, postoperative impingement, and asymptomatic pubic nonunion. Other relatively low incidences but that have severe consequence complications, such as sciatic nerve dysfunction, intraarticular osteotomies, femoral head necrosis, posterior column discontinuity, deep vein thrombosis, major arterial thrombosis, and pulmonary embolism, have been reported [5, 14–17]. During clinical practice, we noticed that some patients with DDH complained of anterior knee pain (AKP) before and after PAO surgery. Previous studies have reported that patients with hip dysplasia also have knee anatomical deformities [18–20]. Therefore, this study intends to analyze the anatomical characteristics of the knee joints of DDH patients and the impact of PAO surgery on the patellofemoral joint (PFJ).

## Methods

Our institutional review board approved the study protocol. Informed consent was obtained from all patients. All investigations were conducted in conformity with ethical principles of research. The patients classified as Hartofilakidis type I DDH who underwent hardware removal surgery after PAO from January 2016 to July 2018 were retrospectively reviewed as the DDH group. The exclusion criteria for the DDH group included knee surgery history, neuromuscular disease, knee varus or valgus, and leg length discrepancy. Patients with PAO combined with femoral osteotomy were also excluded from the DDH group. The sample size for a power of ~ 0.9 (α = .05) indicated a minimum sample size of 78. During the same follow-up period, patients who underwent CT scans at our center due to other diseases were included in the control group. The exclusion criteria for the control group included a history of AKP, peri-knee fractures, ruptured ligaments of knee joints, and Kellgren-Lawrence grade II or more serious OA.

All DDH patients underwent simultaneous CT scans of the hip and knee joints before PAO and after hardware removal surgery, respectively. CT scans were obtained with a Siemens 64-channel scanner (Siemens Healthcare, Munich, Germany). The scanning parameters were as follows: 120 kV, 300 mAs, 512 × 512 matrix, pitch 0.7539, field of view (FOV) 300–400 mm, and slice thickness 0.75 mm (hip) and 2 mm (knee).

The distance from the anterior inferior iliac spine (AIIS) to the ilioischial line (Kohler line), named the DAI was measured in DDH patients. Imaging parameters of knees, including the sulcus angle (SA), femoral trochlear depth (FTD), patellar width (PW), tibial tuberosity-trochlear groove (TT-TG), patellar tilt angle (PTA) and lateral shift of the patella (LSP) were measured in patients in both the DDH and control group. TT-TG, PTA, and LSP of DDH patients were measured before PAO and after hardware removal.

**Fig. 1:**
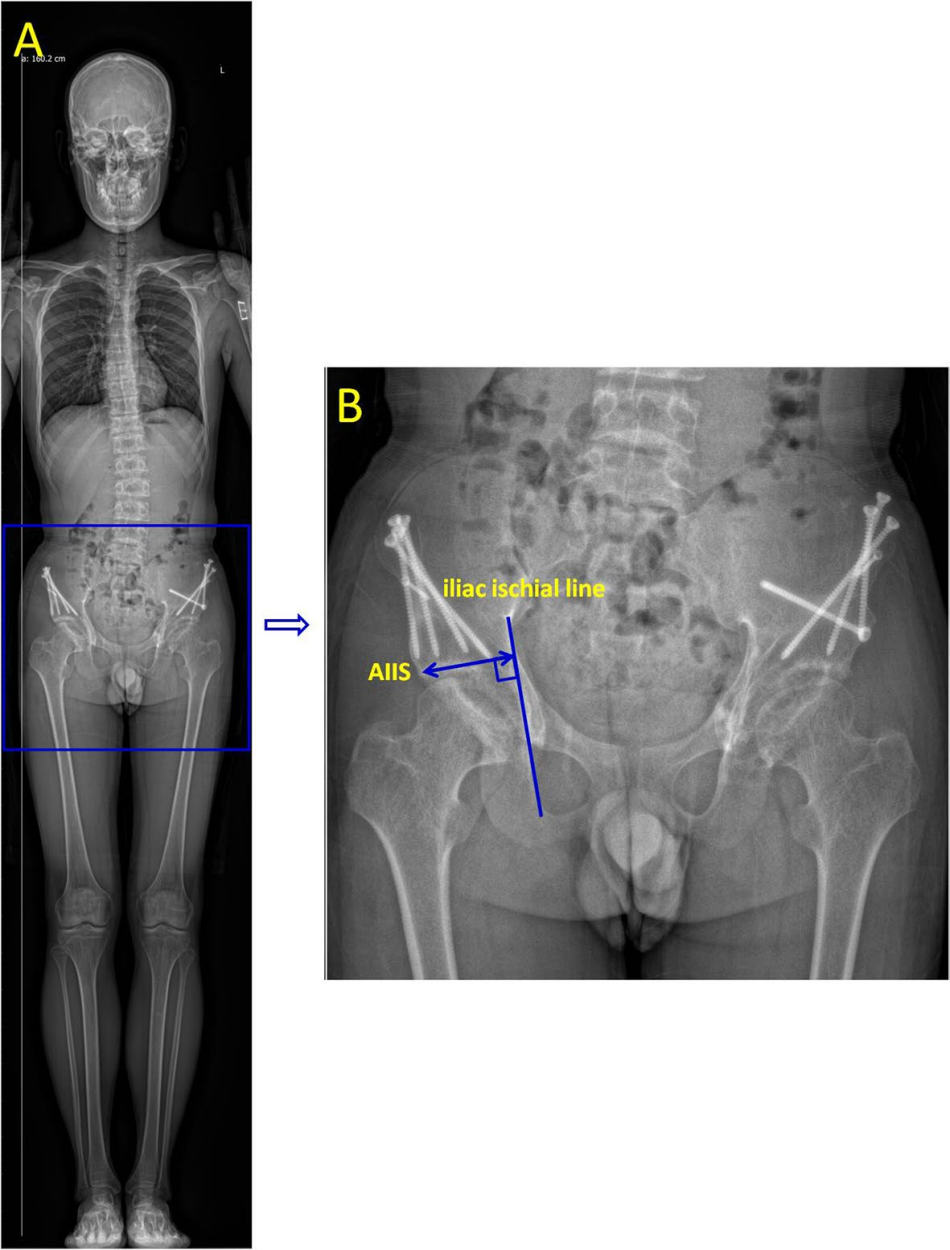
A perpendicular line has been drawn from the AIIS to the iliac ischial line. The distance from the AIIS to the iliac ischial line was measured. The postoperative distance minus the preoperative distance is the displacement of the AIIS (DAIIS). AIIS: anterior inferior iliac spine

**Fig. 2:**
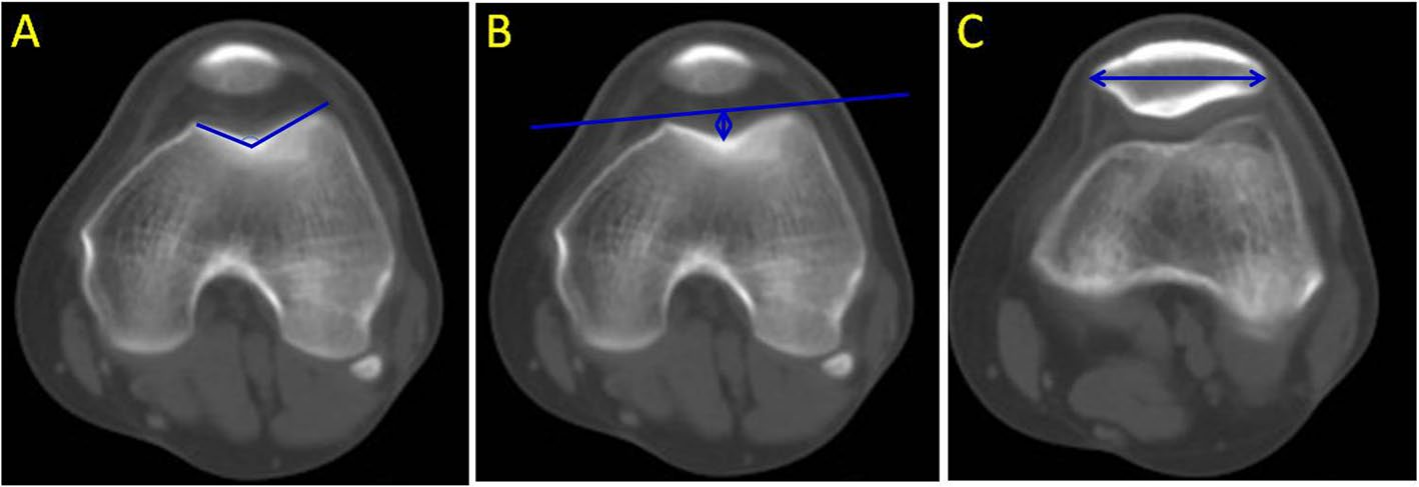
**A** and **B** show the transverse sections passing through the largest femoral condyle. **A** illustrates the sulcus angle (SA). Two lines from the medial and lateral condyles to the deepest point of the intercondylar sulcus formed the SA. **B** illustrates the femoral trochlear depth (FTD). The distance from the deepest point of the femoral trochlear groove to the line connecting the most anterior points on the medial and lateral femur condyles is the FTD. **C** shows the transverse section passing through the midsection of the patella. The distance between the lateral and media of the patella is the patellar width (PW)

**Fig. 3:**
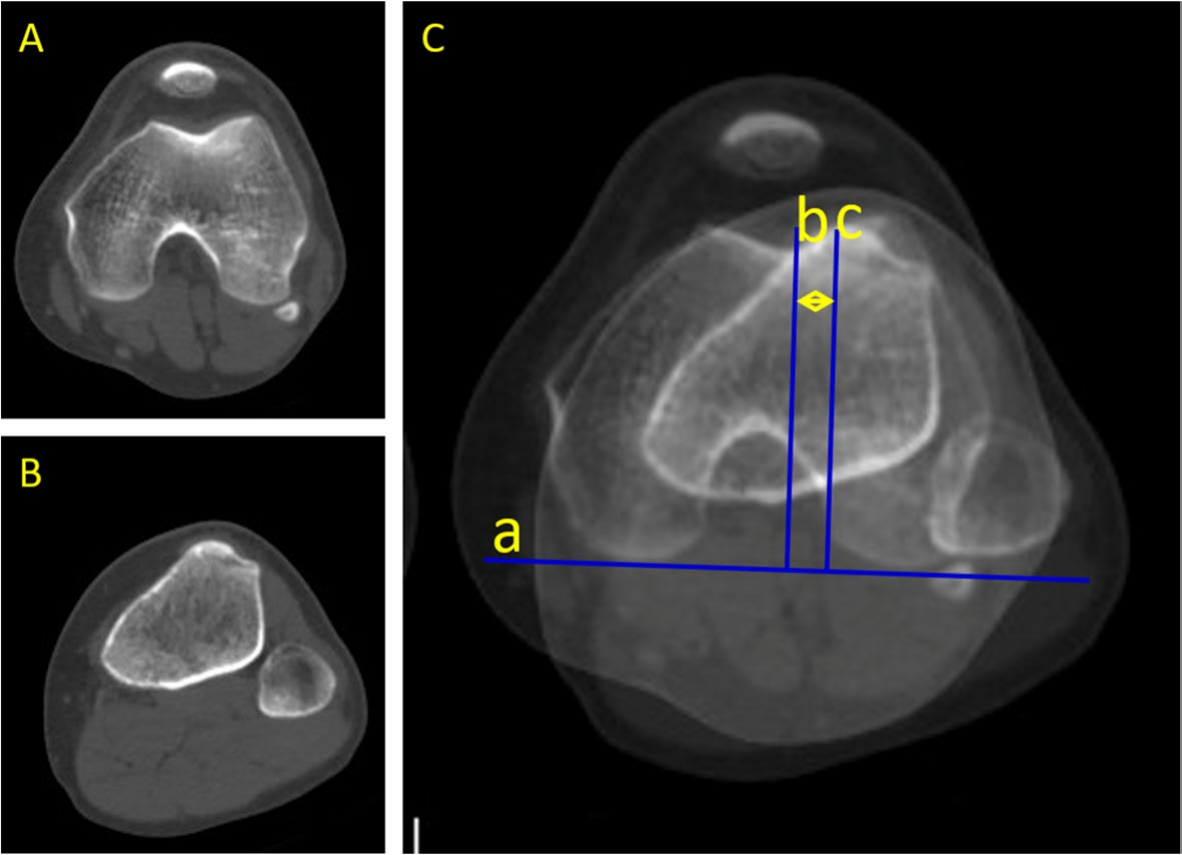
Two transverse images were superimposed to measure the tibial tuberosity-trochlear groove (TT-TG) distance. The first transverse image that depicted a complete cartilaginous trochlea was adopted to determine the deepest point within the trochlear groove. The second transverse image shows the most anterior portion of the tibial tubercle. Line “a” is tangent to the posterior epicondyle. Line “b” goes through the deepest point of the trochlear groove, perpendicular to line “a”. Line “c” is parallel to line “b” and goes through the most anterior portion of the tibial tubercle. The TT-TG measurement is the distance between lines “b” and “c”

**Fig. 4:**
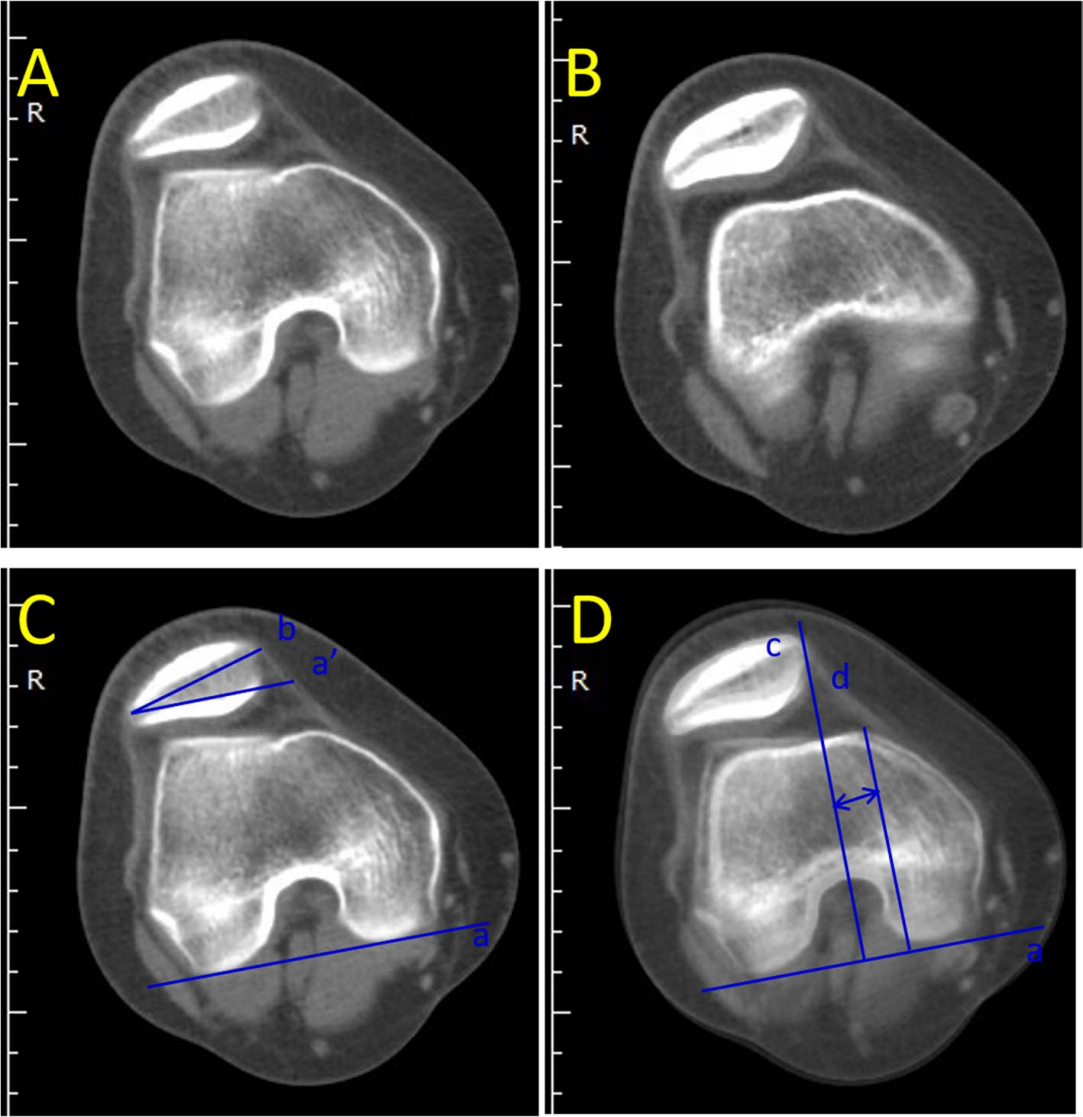
Two transverse images were superimposed to measure the patellar tilt angle (PTA) and the lateral shift of the patella (LSP). The first image depicts the transverse section passing through the largest posterior femoral condyle. The second image shows the transverse section passing through the midsection of the patella. Line “a” is tangent to the posterior epicondyle. Line “b” goes through the horizontal axis of the patella. Line “c” goes through the medial point of the patella and is perpendicular to the line “a.” Line “d” is the perpendicular line from the apex of the medial femur condyle to line “a.” The angle formed by lines “a” and “b” is the PTA. The LSP is the distance between line “c” and line “d”

The specific measurement method was as follows: A vertical line was drawn from the AIIS to the ilioischial line, and the distance from the AIIS to the ilioischial line was the DAI (Fig. 1). The SA and FTD were obtained in transverse sections passing through the largest femoral condyle (Fig. 2) [21]. The SA was defined by two lines drawn from the medial and lateral condyles to the deepest point of the intercondylar sulcus (Fig. 2A). The distance from the deepest point in the femoral trochlear groove to the line connecting the most anterior points on the medial and lateral femur condyles was defined as FTD (Fig. 2B). The TT-TG distance, shown in Fig. 3, was measured according to the method reported by Schoettle [22] and Koëter [23]. The first transverse image that depicted a complete cartilaginous trochlea was adopted to determine the deepest point within the trochlear groove. A line was drawn through the deepest point of the trochlear groove, perpendicular to the posterior condylar tangent line. The second line was parallel to the first line through the most anterior portion of the tibial tubercle. The distance between the two lines represents the TT-TG distance. The PTA was defined by calculating the angle of the two lines. The first line went through the most comprehensive transverse portion of the patella. The second line connected the posterior borders of both posterior femoral condyles. The angle formed between these two lines is the PTA [24, 25]. Fulkerson et al. reported that the posterior condylar line is a more reliable reference point and that it produced much less variable patellar tilt measurements; posterior femoral condyles were used instead of the anterior femoral condyles in this study [26]. The PW was measured in the same image. The distance between two parallel lines in the axial plane defined the LSP, as shown in Fig. 4. The first line is the perpendicular line from the apex of the medial condyle of the femur to the tangent line of the posterior condyle. The second line is the perpendicular line from the medial edge of the patella to the tangent line of the posterior condyle. If LSP is greater than 5 mm, it is defined as lateral patella shift [18].

### Statistical Analysis

Shapiro-Wilk test was used to test for normal distribution. The DAI, PTA, LSP and TT-TG of all DDH patients before and after Bernese PAO were compared using paired t-tests. The FTD, PW, and SA of the DDH patients and the control group were analyzed using independent t-tests. PTA, TT-TG, and LSP between the control group and preoperative DDH patients who were to undergo PAO and between the control group and postoperative DDH patients who underwent PAO were compared using paired t-tests. The proportion of patients with TT-TG greater than 20 mm before and after PAO was analyzed using the Chi-Squared Test. Statistical analyses were conducted using SPSS (version 24.0). Significance was determined at a *p*-value of < 0.05.

## Results

Seventy patients (86 hips) were included in the DDH group of this study. Sixteen of these patients were affected bilaterally. There were 52 females and 18 males, and the mean age was 27.10 ± 9.88 years old. Thirty-three patients (33 knees) were included in the control group. There were 15 men and 18 women, with an average age of 35 ± 24.

A total of 8.57% (6/70) and 22.86% (16/70) of DDH patients complained of AKP pre- and post-PAO, respectively. Of the 70 patients with DDH, two had bilateral AKP after PAO surgery.

The DAI changed from 4.04 ± 0.61 mm before PAO surgery to 5.44 ± 0.63 mm after PAO surgery. The difference was significant (*p*<0.05). The SA, PW, and FTD of the DDH group and control group are shown in Table 1. The SA of the DDH group was greater than that of the control group. The FTD of the DDH group was smaller than that of the control group. Both of these differences were significant (*p*<0.05). The PW of the DDH group was smaller than that of the control group; however, the difference was not statistically significant (*P* = 0.16).

**Table 1:** FTD (mm), SA (degree), and PW(cm) of DDH patients and control group

	DDH	Control	T value	*p* value
FTD	5.45 ± 1.59	7.39 ± 1.20	6.30	*P*<0.05
SA	140.69 ± 11.30	130.82 ± 6.43	4.70	*P*<0.05
PW	4.16 ± 0.36	4.24 ± 0.38	1.06	*P* = 0.16

*FTD* Femoral trochlear depth, *SA* Sulcus angle, *PW* Patellar width

The LSP, the PTA, and the TT-TG of the control group and the DDH patients are shown in Table 2. The changes in these three groups of parameters before and after surgery were not statistically significant. Moreover, either before or after surgery, there was no statistically significant difference in the parameters of these three groups compared with the control group. The proportion of patients with TT-TG greater than 20 mm before and after surgery was 11.63% (10/86) and 9.3% (9/86), respectively. This difference was also not statistically significant.

**Table 2:** LSP (mm), PTA (degree), and TT-TG (mm) of DDH patients and control group pre- and post-op

	DDH		Control	pre-op VS control		post-op VS control		pre-op VS post-op	
	pre-PAO	post-PAO		T value	*p* value	T value	*p* value	T value	*p* value
LSP	5.11 ± 3.39	4.94 ± 3.89	3.78 ± 2.79	2.00	0.06	1.54	0.13	2.09	0.64
PTA	10.68 ± 6.13	10.53 ± 6.46	9.57 ± 4.75	0.92	0.3	0.87	0.38	1.89	0.97
TT-TG	13.88 ± 6.10	13.55 ± 5.75	12.41 ± 4.12	1.26	0.14	1.03	0.24	2.69	0.6

*LSP* Lateral shift of patella, *PTA* Patellar tilt angle, *TT-TG* Tibial tuberosity-trochlear groove

## Discussion

Bernese PAO is the preferred joint-preserving treatment choice of surgeons treating DDH in adults. Significant complications were noted in 6 to 37% of the procedures, according to Clohisy’s review [14]. In our clinical center, we found that in addition to complications already reported in the literature, AKP was an essential factor affecting quality of life and patient satisfaction. DDH patients were followed up in this study; regarding walking on flat ground, squatting, stair climbing and descent, 8.57% of DDH patients had AKP in addition to hip discomfort before PAO surgery, and 22.86% of DDH patients complained of AKP 6 months after surgery. Therefore, in this study, we analyzed the anatomical characteristics of the knee joint in patients with DDH and the effects of PAO surgery on the knee joint, especially the PFJ.

Trochlear dysplasia is characterized by an increased SA and decreased groove depth of the trochlear groove [27, 28]. Dysplasia of the femoral trochlea reduced the lateral stability of the patella by up to 70%, according to Senavongse and his colleagues’ study [29]. It is, therefore, easy to understand that a flat trochlea without a prominent lateral facet lacks a necessary restraint that can predispose the patella to lateral displacement. In the patients followed up in this study, the SA and FTD were different between the DDH group and the control group (*p*<0.05). These anatomical differences could affect PFJ stability and be linked to the occurrence of AKP. Patellar morphology, especially PW, is another factor that causes abnormal stress on the PFJ. The smaller PW also reduces the contact surface of the PFJ, which induces more stress on the PFJ surface. Salsich [25] reported that in pain-free subjects, PW was the main predictor of contact area, explaining 31% of the variance in contact area. The PW of DDH patients in this study was not significantly smaller than that of the control group (*p* = 0.16).

In addition to osseous structures, the direction and magnitude of force produced by the quadriceps muscle have significant influence on PFJ biomechanics [30]. Since being described for the first time by Brattstrom in 1964 [31], Q angle has become an essential parameter to assess the quadriceps muscle lateralization force. The Q-angle was formed by two lines encountered on the center of the patella. One starts at the anterosuperior iliac spine and continues to the center of the patella, and another goes from the tibial tuberosity to the center of the patella [31, 32]. A larger Q-angle represents a higher lateralization force on the patella, which increases the pressure between the lateral patella facet and the lateral femoral condyle [33]. However, some authors have doubted whether the clinical Q-angle can represent the real “line-of-application of quadriceps force” [34, 35]. Not all four muscles that make up the quadriceps are related to the AIIS. The vastus lateralis, vastus medialis, and vastus intermedius all arise from the surface of the femur. The rectus femoris originates from the AIIS with a direct tendon. The reflected tendon attaches to the hip joint capsule anteriorly and to the upper rim of the acetabulum. Since the acetabular fragment was reoriented anterolaterally, PAO surgery resulted in a spatial displacement of the AIIS. In this study, the distance between the AIIS and the ilioischial line (Kohler line) changed significantly post-PAO (*p*<0.05). The distance represents lateralization of the origination point of the rectus femoris. The lateral displacement of the AIIS inevitably leads to an increase in the lateral vector of the quadriceps to the patellofemoral, which in turn increases compressive forces between the lateral facet of the patella and the lateral femoral condyle. This change may partly explain the increase in the number of patients who complained of AKP after PAO surgery. On the other hand, it should be emphasized that acetabular fragment reorientation has a three-dimensional effect on the origin of the rectus femoris. However, this study only considered lateral displacement measurements of the lateral coronal plane.

AKP has been demonstrated as the result of increased PFJ stress due to decreased contact area [30, 33]. In patella positioned more laterally, shift and tilt have been regarded as the main reasons for decreased PFJ contact area. Therefore, does the displacement of AIIS after PAO surgery affect the PFJ? In theory, the lateral displacement of the rectus femoris origin increases the lateral displacement and rotation of the patella. However, in this study, PAO surgery did not significantly affect the LPS or the PTA. One issue that we need to address to but have not yet resolved is whether changes in gait after PAO also affect the PFJ. DDH patients showed hip instability because of the decreasing coverage of the acetabulum to the femoral head. Compensatory pronation gait can increase the stability of the hip joint to a certain extent. However, the possible gait changes after PAO surgery may counteract the effect of the displacement of the AIIS on the lateral displacement and rotation of the patella. Thus, further research is needed to confirm this point.

The TT-TG distance is another essential parameter for the evaluation of patellofemoral instability [36]. It represents a lateralized insertion of the patellar tendon relative to the deepest part of the trochlear groove. The TT-TG distance represents the radiographic measurement of the quadriceps vector, which represents a lateral force-displacement on the patella during knee motion. Although different studies reported different normal values, there was a general consensus that more than 15 mm was considered abnormal, and more than 20 mm was regarded as a significant risk factor for patellar instability [35, 36]. For DDH patients in this study, the TT-TG changed from 13.88 ± 6.10 mm pre-PAO to 13.55 ± 5.75 mm post-PAO. This difference was not statistically significant. Both preoperative and postoperative TT-TG were higher than that of the control group (12.41 ± 4.12 mm), but the difference was not statistically significant. It should be noted that the proportion of patients with TT-TG higher than 20 mm was 11.63% pre-PAO and 9.3% post-PAO. If this part of the patient group complained of AKP, surgical correction by osteotomy and subsequent medial transfer of the tibial tubercle should be considered, but this approach requires further in-depth study.

There were several limitations to our study that need to be mentioned. First, there were fewer cases in the control group, and there were no pelvic films in the control group. Therefore, it was impossible to exclude DDH patients from the control group. However, no patients complained of a history of hip and knee discomfort in the control group. Second, this study mainly focused on the skeletal structure characteristics of PFJs. However, the skeletal structure only provided static stabilization. Muscles, ligaments, and retinacula provide active and passive stabilization. Third, PFJ dysfunction is a complex issue. Changes in gait post-PAO in DDH patients also affects the PFJ, but this study has not yet analyzed gait changes; hence, gait changes need to be further studied. Finally, tibial rotation, varus, and valgus deformities of the knee could also affect patellar tracking and the stability of the PFJ. However, due to the limitation of image data in this study, these potential factors were not included.

## Conclusion

The knee joints of DDH patients presented a certain degree of femur trochlear groove dysplasia. PAO surgery did not change PFJ stability, although the origination point of the rectus femoris muscle moved laterally during PAO surgery. For patients with DDH, the symptoms and deformities of the knee joint should be considered at the same time as a hip osteotomy surgery program. Simultaneous surgeries to improve the PFJ biomechanical environment may improve the quality of life of DDH patients who complain of AKP before PAO. We also must acknowledge that PFJ disorder is a complex issue, and it is difficult to form a consensus on its treatment. The indications for these operations for PFJ need to be further clarified.

## Data Availability

The datasets used and/or analyzed during the study are available from the corresponding author upon reasonable request.

## Abbreviations

PAO: Periacetabular osteotomy
DDH: Developmental dysplasia of the hip
AKP: Anterior knee pain
PFJ: Patellofemoral joint
DAI: Distance from the anterior inferior iliac spine to the ilioischial line
CT: Computed tomography
SA: Sulcus angle
FTD: Femoral trochlear depth
PW: Patellar width
TT-TG: Tibial tuberosity-trochlear groove
PTA: Patellar tilt angle
LSP: Lateral shift of the patella
OA: Osteoarthritis
HHS: Harris hip score
LFCN: Lateral femoral cutaneous nerve
